# An *In Silico* Analysis Reveals an EMT-Associated Gene Signature for Predicting Recurrence of Early-Stage Lung Adenocarcinoma

**DOI:** 10.1177/11769351221100727

**Published:** 2022-05-23

**Authors:** Yi Han, Fang Cheng Wong, Di Wang, Christoph Kahlert

**Affiliations:** 1 Department of Visceral, Thoracic and Vascular Surgery, University Hospital and Faculty of Medicine Carl Gustav Carus, Technische Universität Dresden, Dresden, Germany; 2 Department of Respiratory Medicine, Fourth Affiliated Hospital, Harbin Medical University, Harbin, China

**Keywords:** Early-stage lung adenocarcinoma, recurrence, epithelial-mesenchymal transition, GEO, gene signature

## Abstract

**Background:**

The potential micrometastasis tends to cause recurrence of lung adenocarcinoma (LUAD) after surgical resection and consequently leads to an increase in the mortality risk. Compelling evidence has suggested the underlying mechanisms of tumor metastasis could involve the activation of an epithelial-mesenchymal transition (EMT) program. Hence, the objective of this study was to develop an EMT-associated gene signature for predicting the recurrence of early-stage LUAD.

**Methods:**

The mRNA expression data of patients with early-stage LUAD were downloaded from Gene Expression Omnibus (GEO) and The Cancer Genome Atlas (TCGA) available databases. Gene Set Variation Analysis (GSVA) was first performed to provide an assessment of EMT phenotype, whereas Weighted Gene Co-expression Network Analysis (WGCNA) was constructed to determine EMT-associated key modules and genes. Based on the genes, a novel EMT-associated signature for predicting the recurrence of early-stage LUAD was identified using a least absolute shrinkage and selection operator (LASSO) algorithm and a stepwise Cox proportional hazards regression model. Kaplan-Meier survival analysis, receiver operating characteristic (ROC) curves and Cox regression analyses were used to estimate the performance of the identified gene signature.

**Results:**

GSVA revealed diverse EMT states in the early-stage LUAD. Further correlation analyses showed that the EMT states presented high correlations with several hallmarks of cancers, tumor purity, tumor microenvironment cells, and immune checkpoint genes. More importantly, Kaplan-Meier survival analyses indicated that patients with high EMT scores had worse recurrence-free survival (RFS) and overall survival (OS) than those with low EMT scores. A novel 5-gene signature (*AGL, ECM1, ENPP1, SNX7*, and *TSPAN12*) was established based on the EMT-associated genes from WGCNA and this signature successfully predicted that the high-risk patients had a higher recurrence rate compared with the low-risk patients. In further analyses, the signature represented robust prognostic values in 2 independent validation cohorts (GEO and TCGA datasets) and a combined GEO cohort as evaluated by Kaplan-Meier survival (*P*-value < .0001) and ROC analysis (AUC = 0.781). Moreover, the signature was corroborated to be independent of clinical factors by univariate and multivariate Cox regression analyses. Interestingly, the combination of the signature-based recurrence risk and tumor-node-metastasis (TNM) stage showed a superior predictive ability on the recurrence of patients with early-stage LUAD.

**Conclusion:**

Our study suggests that patients with early-stage LUAD exhibit diverse EMT states that play a vital role in tumor recurrence. The novel and promising EMT-associated 5-gene signature identified and validated in this study may be applied to predict the recurrence of early-stage LUAD, facilitating risk stratification, recurrence monitoring, and individualized management for the patients after surgical resection.

## Introduction

Lung cancer remains the leading cause of cancer-related death worldwide, with 1,796,144 cases of mortality from both sexes and all ages recorded in 2020.^1^ Among the different subtypes of lung cancer, lung adenocarcinoma (LUAD) as the most common subtype of non-small cell lung cancer (NSCLC), accounts for more than 40% of all types of lung cancer.^2^ Surgical resection is currently the preferred treatment strategy for patients with early-stage NSCLC. Unfortunately, the patients are still at high risk of tumor recurrence after complete resection.^3^ Of those who are amenable to surgical resection, 25% to 30% of patients with stage-I NSCLC still develop local recurrence or distant metastasis even when the resection results in microscopically negative margins.^4,5^ Moreover, tumor recurrence after surgical resection causes treatment failure, which severely limits the survival of patients and is closely linked to the mortality risk. Low post-recurrence survival in resected stage-I NSCLC has been documented by many reports,^6-8^ suggesting that tumor recurrence significantly raises the risk of death from NSCLC. One of the main causes of tumor recurrence could be associated with the scattered micrometastasis or occult tumor cells in peripheral blood, bone marrow, lymph node or serous cavity even before surgical resection,^9^ which are difficult to be detected by conventional clinical approaches. Hence, the search for a new and effective way to stratify the risk of recurrence for LUAD is thus of central importance.

Epithelial-mesenchymal transition (EMT) is a reversible process by which epithelial cells progressively acquire a range of mesenchymal characteristics, with the ability to invade the tissue surrounding primary tissue, intravasate and eventually enter the circulation.^10^ During the development and progression of many different types of carcinomas, including lung cancer, several signaling pathways activate EMT by inducing the expression levels of a wide variety of EMT transcription factors (EMT-TFs),^11^ thereby promoting tumor migration, invasion, and metastasis. Circulating tumor cells (CTC) exhibit distinct epithelial and mesenchymal phenotypes,^12^ indicating that the EMT process has been activated during the dissemination of tumor cells. Upon arrival at secondary sites, mesenchymal cells can regain the epithelial phenotype by activating epithelial-related gene machinery, which is a process known as mesenchymal-epithelial transition (MET) to complete the colonization process.^13^ It is this exchange program between EMT and MET that allows tumor cells to invade and disseminate to the surrounding and/or distant sites. At the cellular level, EMT occurs through distinct intermediate hybrid states in tumors, which has been proven to be not just a simple binary process but a plastic continuum of partial EMT states between the epithelial and mesenchymal forms,^14-17^ resulting in intratumoral heterogeneity and displaying differences in stemness, tumor-initiating ability, invasiveness, and drug resistance.^18,19^ Therefore, considering the multi-faceted impacts of EMT on tumor progression, we aim to explore a robust EMT-related signature for predicting the recurrence of early-stage LUAD.

## Methods

### Microarray data collection and processing

Two LUAD datasets from Gene Expression Omnibus (GEO) database (https://www.ncbi.nlm.nih.gov/geo/), GSE31210 and GSE50081 were selected for this study. The mRNA expression data and clinical information were downloaded. The selection criteria were as follows: i) gene expression profiling was performed on the same chip platform; ii) the LUAD tumor samples were resected at early stages (stage I or II); iii) the recurrence time of patients were >30 days to avoid complications from the surgery that are not related to the cancer; iv) the availability of the clinical survival information. The microarray datasets of GSE31210 and GSE50081 performed on Affymetrix Human Genome U133 Plus 2.0 Array were then downloaded for subsequent analysis. GSE31210 consisted of 226 patient samples and GSE50081 was made up of 124 patient samples. The clinical features of all the patients including age, gender, smoking status, tumor stage and the follow-up time were presented in Supplemental Table S1. Recurrence-free survival (RFS) is defined as “the time from date of resection surgery to the time of recurrence or death,” whereas overall survival (OS) refers to “the time from date of resection surgery to the time of death.” Since GSE31210 dataset contained a higher number of patient samples, it was used as a training cohort, while the other GSE50081 dataset was used as an internal validation cohort. The mRNA expression data from GSE31210 and GSE50081 were processed *via* affy package in R 4.0.2 and normalized *via* the robust multi-array average (RMA) algorithm. Probe IDs were annotated using the annotation package and the gene symbol with the highest expression value remained. To increase the reliability and accuracy of the data analysis, the sva package in R 4.0.2 was applied to remove the batch effects between GSE31210 and GSE50081 datasets. Additionally, a LUAD dataset (n = 307) from The Cancer Genome Atlas (TCGA) was used as an external validation cohort, and the fragments per kilobase of transcript per million fragments mapped reads (FPKM) values and clinical data were acquired from Genomic Data Commons using UCSC Xena browser (https://xenabrowser.net/datapages/). The HALLMARK gene sets were downloaded from Molecular Signatures Database v7.2 (https://www.gsea-msigdb.org/gsea/index.jsp).

### Gene set variation analysis (GSVA) and correlation analysis

GSVA enrichment scores are generated based on gene expression of each sample by the GSVA algorithm, including gene expression level statistic, rank order per sample, Kolmogorov- Smirnov like random statistic and different score distributions.^20^ Enrichment scores of the 50 HALLMARK gene sets (including EMT gene set) were calculated using the GSVA package in R 4.0.2 for each sample in GSE31210 and GSE50081. EMT enrichment scores were then extracted and used for further analyses. The tumor purity was calculated using the estimate package in R 3.6.1 and tumor microenvironment (TME) cell estimation was performed using the ConsensusTME package in R 4.0.2.^21^ After that, Pearson correlations were calculated between enrichment scores of the 50 HALLMARK gene sets. As for Spearman correlations, the EMT score from each patient was used to calculate the correlations with tumor purity, TME cells and the expression of immune checkpoint genes in the 2 cohorts. The correlations of EMT scores with RFS and OS were analyzed using Kaplan-Meier method and log-rank test. The survival curves were performed using the survival and survminer package in R 4.0.2. *P* < .05 was considered statistically significant.

### Construction of weighted gene co-expression network analysis (WGCNA) and function analysis of key genes

To identify EMT-associated key genes for a signature establishment, WGCNA was constructed^22^ using GSE31210 cohort as GSE31210 dataset contained more patient samples than GSE50081. Here, the mean absolute deviation (MAD) of each gene was calculated in the expression matrix, and the top 5000 genes with the highest MAD were chosen for WGCNA using the WGCNA package in R 4.0.2. In this part, a soft thresholding power was selected to ensure a scale-free network. Under this condition, a co-expression network was constructed by the blockwiseModules function of the WGCNA package. The minimum number of genes in modules was set as 50. Subsequently, the corresponding modules were obtained. The most relevant module to EMT phenotype was selected as the key module, from which the key genes were extracted. Gene Ontology (GO) and Kyoto Encyclopedia of Genes and Genomes (KEGG) pathway enrichment analyses were performed using the Database for Annotation, Visualization, and Integrated Discovery (DAVID, https://david.ncifcrf.gov/home.jsp). Protein-protein interaction (PPI) network was constructed to reveal the relationship among the EMT-associated key genes by Search Tool for the Retrieval of Interacting Genes/Proteins (STRING, http://stringdb.org/)^23^ and Cytoscape_3.8.1.

### Identification and validation of gene signature

Based on the EMT-associated genes from WGCNA, a univariate Cox proportional hazard regression analysis was conducted to screen and identify the genes significantly correlated with RFS in GSE31210 (*P* < .01 as selection criteria) using the survival package in R 4.0.2. The least absolute shrinkage and selection operator (LASSO) algorithm was applied in a Cox regression model to further select the variables by 10-fold cross-validation using the glmnet package in R 4.0.2. Subsequently, an EMT-associated gene signature was built *via* a stepwise Cox proportional hazards regression model. The risk score of the signature for each patient was calculated according to the expression of each gene in the signature and resulting Cox regression coefficients as follows: risk score =  (Gene1 Exp * Gene1 Coef) +  (Gene2 Exp * Gene2 Coef) + (Gene3 Exp * Gene3 Coef) + (Gene4 Exp * Gene4 Coef) + *. . .*. Consequently, an optimal cutoff score was acquired using survminer package in R for RFS analysis, by which the patients were divided into a high-risk group and a low-risk group. Finally, the predictive performance and clinical independence were evaluated by performing Kaplan-Meier survival analysis, ROC curves, and univariate and multivariate Cox regression analyses.^24,25^ *P* < .05 was considered statistically significant. For validation, patients from GSE50081 and TCGA LUAD datasets were similarly assigned into 2 groups: high-risk and low-risk, by calculating the risk scores with the previous formula and using the same cutoff. In addition, the predictive performance of our EMT-associated 5-gene signature was also compared with that of 2 other published signatures.

## Results

### EMT positively correlates to fibroblasts, recurrence-free survival and overall survival rates of patients with early-stage LUAD

**Figure 1.**
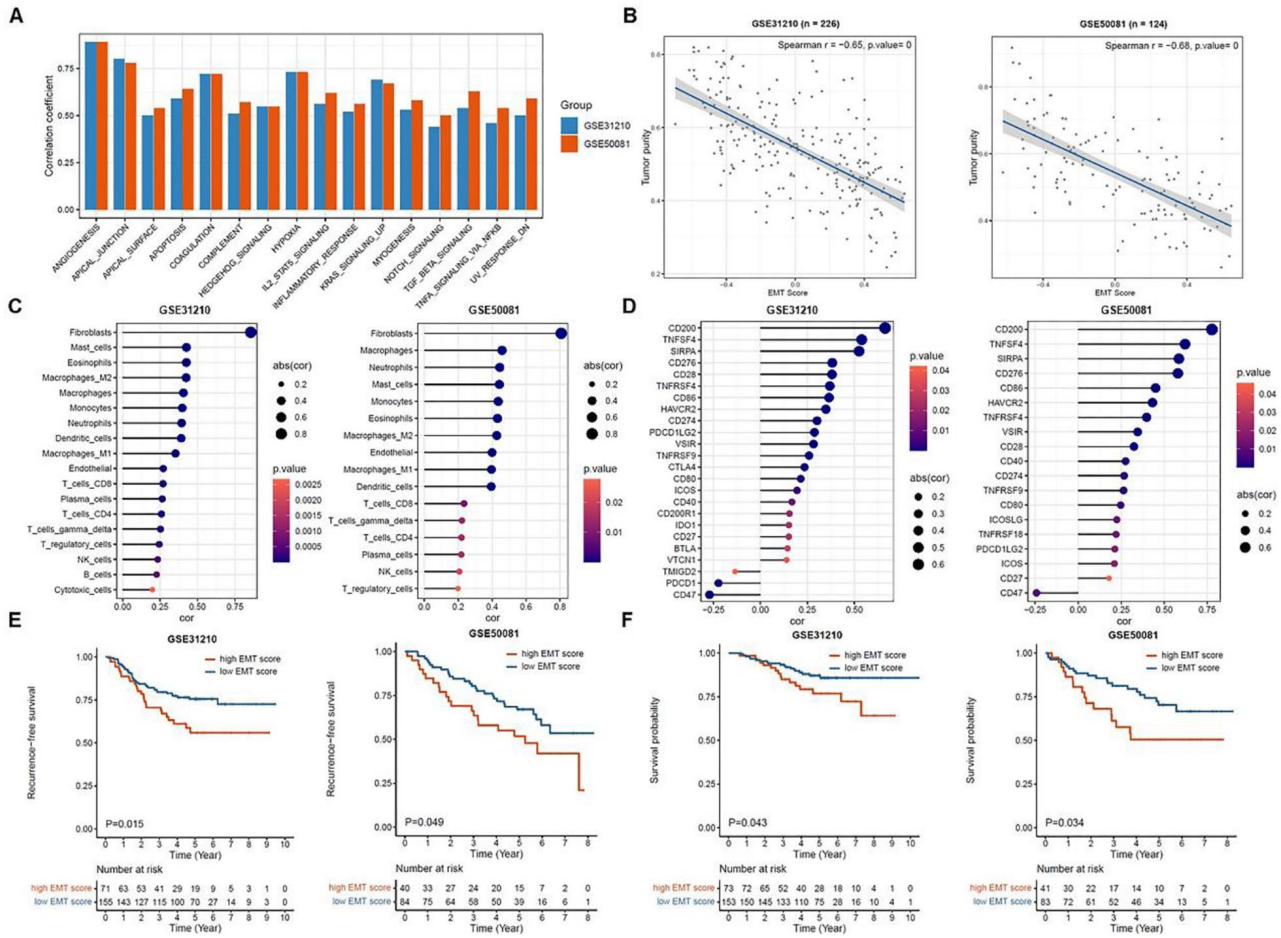
Correlation analyses of EMT in GSE31210 and GSE50081: (A) Pearson correlations of EMT with the HALLMARK gene sets (*P* < .05), (B–D) Spearman correlations of EMT with tumor purity, TME cells and immune checkpoint genes, and (E and F) Kaplan–Meier analyses of RFS and OS based on EMT scores.

As a basis for further understanding the EMT in early-stage LUAD, GSVA was first performed in 2 GEO cohorts (GSE31210 and GSE50081) to calculate the enrichment scores for EMT analysis. The results showed that EMT displayed moderately strong positive correlations (correlation coefficient > .5) with gene sets involved in the cellular processes such as angiogenesis, apical junction, hypoxia, coagulation, KRAS signaling, apoptosis and transforming growth factor beta (TGFβ) signaling (Figure 1A). Pearson correlations between all the gene sets with *P* < .05 were shown in Supplemental Figure S1. Since the investigation of EMT concerns tumor cells, the correlation between tumor purity and EMT was evaluated. Interestingly, the results demonstrated that EMT were significantly and negatively correlated to tumor purity (*r* = −0.65 for GSE31210; *r* = −0.68 for GSE50081) (Figure 1B). As most expression profiling uses bulk clinical specimens, this negative correlation between tumor purity and EMT may indicate that there could be the presence of stromal cells, which also display EMT canonical markers. Considering TME and immune checkpoints play critical roles in the tumor progression, correlations between EMT and TME cells as well as immune checkpoint genes were determined. Figure 1C illustrated the correlations between EMT and 18 types of TME cells, especially a significantly strong positive correlation with fibroblasts (*r* = .86 in GSE31210; *r* = .81 in GSE50081). Next, 31 immune checkpoint molecules shown in Supplemental Table S2 were selected for correlation analyses based on the current literature searches.^26-29^ The analysis revealed that EMT was significantly correlated to cluster of differentiation 200 (*CD200*), tumor necrosis factor ligand superfamily member 4 (*TNFSF4*) and signal regulatory protein alpha (*SIRPA*) in both cohorts (Figure 1D, *P* < .05). To evaluate the correlations of EMT with RFS and OS in the patients with early-stage LUAD, Kaplan-Meier method and log-rank test were used to perform RFS and OS analyses. The patients in the high EMT score group had significantly worse RFS (Figure 1E) and OS (Figure 1F) than those in the low EMT score group. Here, the patients of GSE31210 and GSE50081 cohorts were separated into high and low EMT score groups using the same cutoff for RFS (cutoff = 0.274) or OS (cutoff = 0.265) analyses, respectively. In short, correlation analyses from various aspects showed the connection of EMT between TME cells, immune checkpoints, RFS and OS of patients with early-stage LUAD.

### Construction of weighted co-expression network and identification of key modules

**Figure 2.**
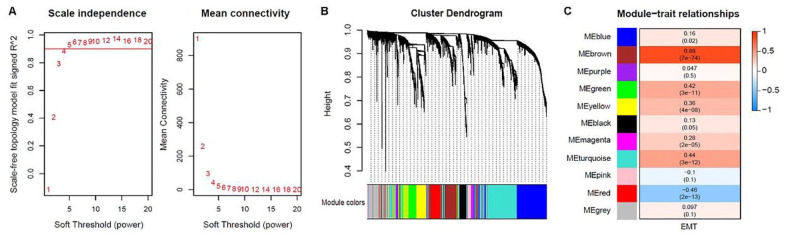
The weighted gene co-expression network analysis: (A) Analysis of scale-free index and mean connectivity for the different soft thresholding powers, (B) Dendrogram of 5000 genes clustered based on the measurement of dissimilarity, and (C) Heatmap of correlations between the module eigengenes and EMT.

To identify key modules and genes involved in the EMT phenotype, gene co-expression networks were constructed based on the expression values of the top 5000 genes with the highest MAD in 226 samples of the GSE31210 cohort (Supplemental Figure S2). Here, the soft thresholding power, β = 4 (scale-free *R^2* = 0.9), was used to construct a scale-free network (Figure 2A). As a result, 10 co-expression modules were identified: module1 (turquoise) with 1261 genes, module 2 (blue) with 985 genes, module 3 (brown) with 591 genes, module 4 (yellow) with 447 genes, module 5 (green) with 345 genes, module 6 (red) with 291 genes, module 7 (black) with 194 genes, module 8 (pink) with 100 genes, module 9 (magenta) with 72 genes, module 10 (purple) with 62 genes (Figure 2B). The results indicated that module 3 (brown) was the most relevant module to EMT phenotype (*r* = 0.88, *P* = *7e-74*), and this module was thus considered the key module for further analysis (Figure 2C).

**Figure 3.**
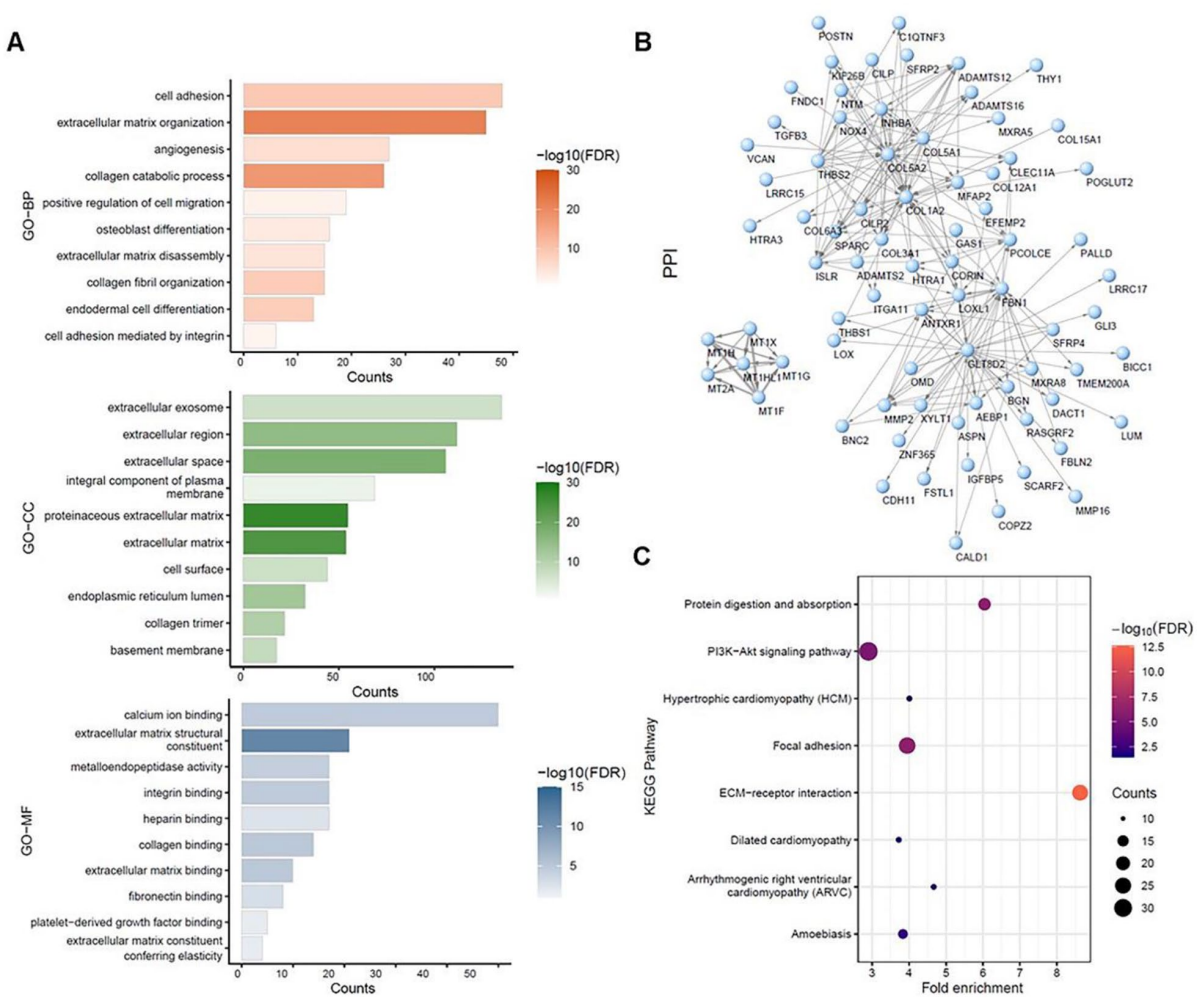
GO, KEGG pathway and PPI network analysis of the genes in brown module: (A) Biological process, Cellular component, and Molecular function analysis (top 10 enrichment results), (B) PPI network analysis. The arrows indicated the directions of action between genes, and the connection widths indicated weight value, representing connection strength between genes (nodes), and (C) KEGG pathway analysis (FDR < 0.05).

To further evaluate the gene functions of the key module, DAVID was used for GO and KEGG analysis. The analysis revealed that genes from module 3, the most relevant to EMT, were involved in biological process (BP) related to cell adhesion, extracellular matrix organization and disassembly, collagen catabolic process, angiogenesis, and positive regulation of cell migration (Figure 3A). As for cellular component (CC), these highly relevant EMT-related genes were found mainly on extracellular exosome, extracellular matrix and basement membrane (Figure 3A). At the cellular level, the molecular function (MF) of the genes from module 3 could be related to extracellular integrin binding, collagen binding, extracellular matrix binding, fibronectin binding, platelet-derived growth factor binding (Figure 3A), which all play vital roles in tumor progression and metastasis. To reveal the relationship among these genes from module 3, the PPI network was constructed by using the top 200 connections with the highest weight value (Figure 3B). The results indicated that the EMT-related genes in module 3 had a close connection. Consistently, KEGG results also showed that these genes from module 3 were highly associated with 8 pathways related to cancer metastasis, such as PI3K-Akt signaling pathway, focal adhesion, ECM-receptor interaction (Figure 3C, false discovery rate (FDR) < 0.05). Taken together, these functional analyses suggest that these genes from module 3 were indeed relevant to EMT since they were involved in cancer migration and invasion cellular processes and pathways.

### Establishment of the EMT-associated gene signature for prediction of recurrence

**Figure 4.**
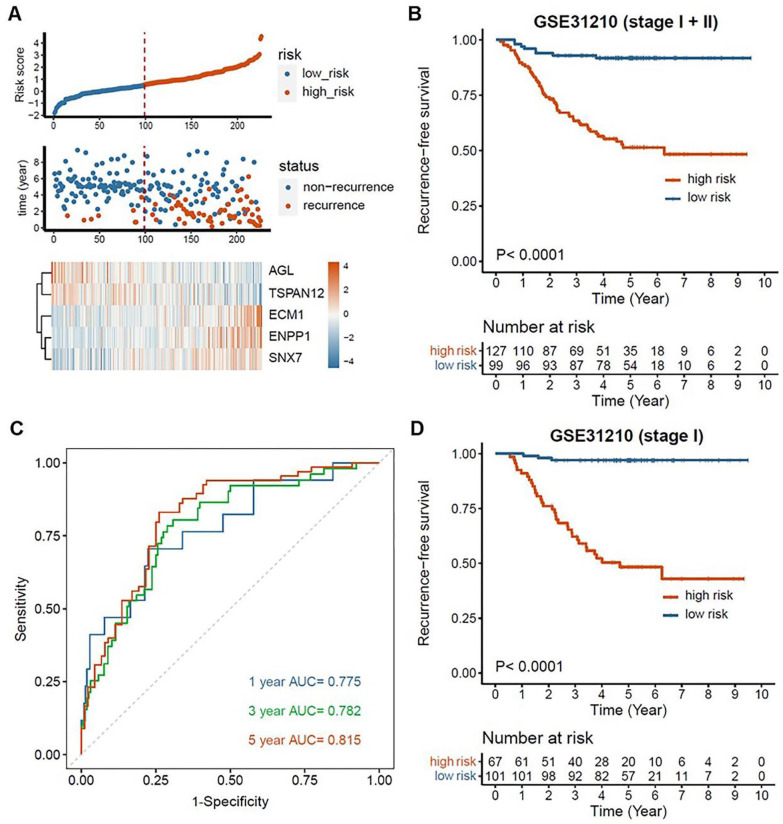
Establishment and evaluation of the EMT-associated gene signature in GSE31210: (A) The distribution of risk score, recurrence time and status and gene expression of the signature, (B) Kaplan–Meier analysis of RFS in early-stage LUAD based on the risk score, (C) Time-dependent ROC analysis of the EMT-associated gene signature for evaluation of predictive performance, and (D) Kaplan–Meier analysis of RFS in stage-I LUAD based on the risk score.

After gaining insights into the possible functions of EMT-associated genes, an EMT-associated gene signature was constructed to predict the recurrence of the patients after early-stage LUAD resection. 187 out of 591 genes were found to be significantly related to RFS based on a univariate Cox proportional hazard regression analysis (Supplemental Table S3, *P* < .01). The LASSO algorithm was used for variable selection in a Cox regression model to enhance the prediction accuracy. The value 0.05542331 was chosen for optimal Lambda using 10-fold cross-validation *via* the minimum criteria. As a result, 11 genes were obtained through LASSO analysis (Supplemental Figure S3 and Table S4) and further used to establish the signature *via* a stepwise Cox proportional hazards regression model. Eventually, 5 of them, that is, Amylo-Alpha-1, 6-Glucosidase, 4-Alpha-Glucanotransferase (*AGL*), extracellular matrix protein 1 (*ECM1*), ectonucleotide pyrophosphatase/ phosphodiesterase 1 (*ENPP1*), sorting nexin 7 (*SNX7*), tetraspanin 12 (*TSPAN12*) were extracted to establish the EMT-associated 5-gene signature (Supplemental Table S5). The risk score of the signature for each patient was calculated as follows: risk score = AGL expression * (−0.488280568)  + *ECM1* expression * 0.269189487 + *ENPP1* expression * 0.26414351 + *SNX7* expression * 0.598307462 + *TSPAN12* expression * (− 0.486050514). In GSE31210 cohort, the patients whose risk scores were scaled between −1.800 and +4.535 were divided into the high-risk group (n = 127) and low-risk group (n = 99) based on the optimal cutoff score (Figure 4A, cutoff = 0.550). Kaplan-Meier survival analysis displayed that the high-risk patients had a higher recurrence rate than the low-risk patients in the early-stage LUAD (Figure 4B, *P* < .0001). The time-dependent ROC curve demonstrated that our signature had a good predictive performance, with values of area under the curve (AUC) of 0.775, 0.782, and 0.815 for 1, 3, and 5 years, respectively (Figure 4C). Moreover, the RFS of the high-risk patients with stage I was shorter than that of the low-risk patients based on Kaplan-Meier analysis (Figure 4D, cutoff = 0.700, *P* < .0001).

### Validation of the EMT-associated gene signature

**Figure 5.**
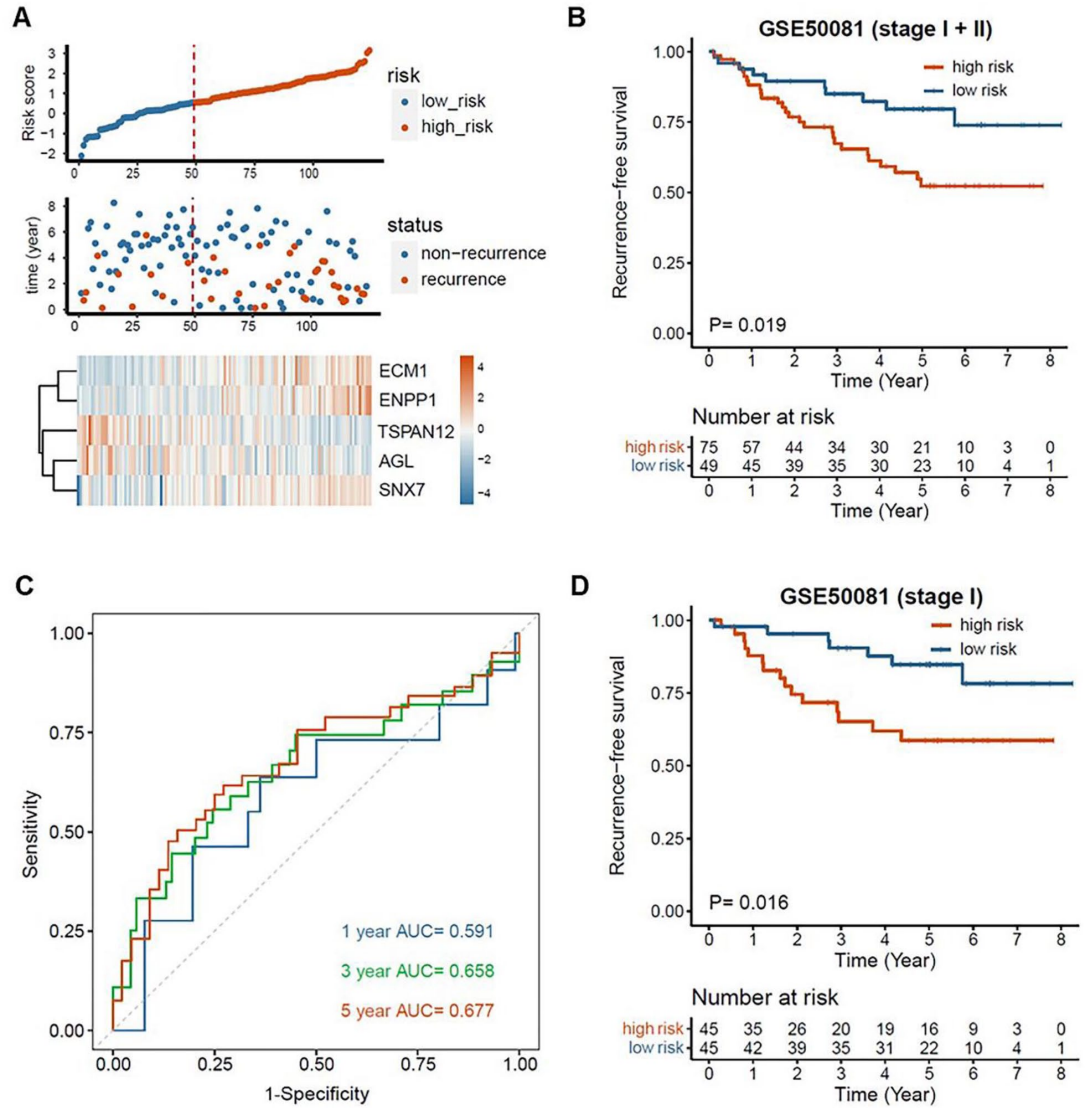
Validation of the EMT-associated gene signature in GSE50081: (A) The distribution of risk score, recurrence time and status and gene expression of the signature, (B) Kaplan–Meier analysis of RFS in early-stage LUAD based on the risk score, (C) Time-dependent ROC analysis of the EMT-associated gene signature for evaluation of predictive performance, and (D) Kaplan–Meier analysis of RFS in stage-I LUAD based on the risk score.

To validate the EMT-associated gene signature, GSE50081 dataset was used as an internal validation cohort. The patients’ risk scores were calculated using the previous formula and were scaled between −2.111 and +3.146. Then the patients were divided into the high-risk group (n = 75) and the low-risk group (n = 49) with the same cutoff value as in GSE31210 (Figure 5A, cutoff = 0.550). It is noteworthy that similar results were obtained in the validation cohort. Kaplan-Meier survival analysis revealed that the high-risk patients had a worse RFS than the low-risk patients in the early-stage LUAD (Figure 5B, *P* = .019). The time-dependent ROC showed AUC values of 0.591, 0.658, 0.677 at 1, 3,and 5 years, respectively (Figure 5C). Furthermore, the stage-I patients with high risk had worse RFS than those with low risk (Figure 5D, cutoff = 0.700, *P* = .016). All the available patient information and risk scores generated from this study for each patient were presented in Supplemental Table S6.

### Clinical independence of the EMT-associated gene signature

To assess the independence of the EMT-associated gene signature in clinical application, univariate and multivariate Cox regression analyses were performed with several clinical characteristics, including age, gender, smoking status and tumor-node-metastasis (TNM) stage. The results displayed the risk score and TNM stage were independent unfavorable factors for RFS in GSE31210 cohort (univariate Cox: stage I/II with *P* < .001 and risk score with *P* < .001; multivariate Cox: stage I/II with *P* = .035 and risk score with *P* < .001) and GSE50081 (univariate Cox: stage I/II with *P* = .006 and risk score with *P* = .010; multivariate Cox: stage I/II with *P* = .027 and risk score with *P* = .045), respectively (Tables 1 and 2).

**Table 1.** Univariate and multivariate Cox regression analyses of the 5-gene signature in GSE31210 cohort.

Variables		Univariate Cox			Multivariable Cox		
		HR	95% CI	*P*-Value	HR	95% CI	*P*-Value
Age	N = 226	1.030	0.997-1.070	.074	1.030	0.993-1.067	.112
Gender	N = 226	0.787	0.482-1.280	.338	1.345	0.658-2.747	.416
Male/female							
Smoking	N = 226	1.330	0.815-2.180	.252	1.181	0.583-2.391	.644
Non-smoker/smoker							
Stage	N = 226	3.160	1.920-5.210	<.001	1.793	1.043-3.080	.035
I/ II							
Risk score	N = 226	2.720	2.130-3.470	<.001	2.538	1.949-3.303	<.001

Abbreviations: 95% CI, 95% confidence interval; HR, hazard ratio.

**Table 2.** Univariate and multivariate Cox regression analyses of the 5-gene signature in GSE50081 cohort.

Variables		Univariate Cox			Multivariable Cox		
		HR	95% CI	*P*-Value	HR	95% CI	*P*-Value
Age	N = 124	0.995	0.964-1.030	.774	0.992	0.961-1.024	.609
Gender	N = 124	0.835	0.437-1.600	.586	0.760	0.383-1506	.431
Male/female							
Smoking	N = 113	0.862	0.405-1.830	.699	0.731	0.331-1.612	.437
Non-smoker/smoker							
Stage	N = 124	2.520	1.300-4.890	.006	2.244	1.098-4.588	.027
I/ II							
Risk score	N = 124	1.610	1.120-2.300	.010	1.466	1.008-2.131	.045

Abbreviations: 95% CI, 95% confidence interval; HR, hazard ratio.

**Figure 6.**
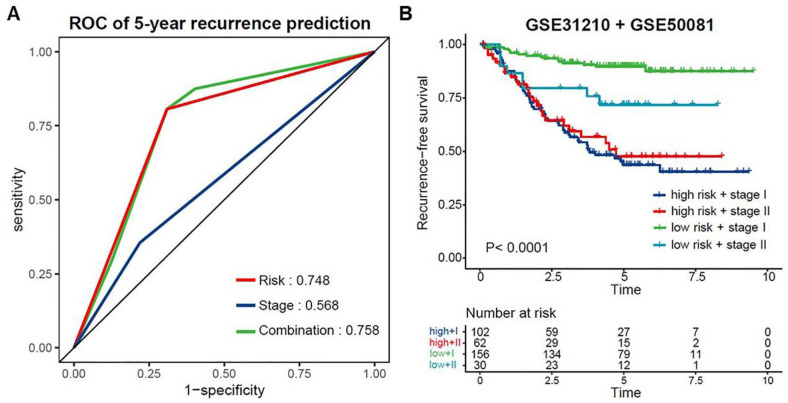
Prediction performance of the signature-based recurrence risk combined with TNM stage: (A) ROC curves for 5-year recurrence prediction of recurrence risk combined with TNM stage in the combined cohort of GSE31210 and GSE50081, and (B) Kaplan–Meier analysis of RFS in 4 subgroups of the combined cohort of GSE31210 and GSE50081.

Considering that the risk score and TMN stage were shown as independent unfavorable factors in LUAD prognosis, we evaluated the combined effect of the 2 factors on the 5-year recurrence prediction in the patients from the combined cohort of GSE31210 and GSE50081 (n = 350). The patients were first divided into a high-risk group (n = 164) and a low-risk group (n = 186) using the optimal cutoff value through the ROC curve and Youden index analysis^30^ (cutoff = 0.851). The ROC curve showed that the combination of the signature-based recurrence risk and TNM stage factors represented a higher prognostic value (AUC = 0.758) for the prediction of 5-year recurrence compared with recurrence risk alone (AUC = 0.748) and stage alone (AUC = 0.568) (Figure 6A), suggesting that these 2 factors provide a better predictive value on patients with early-stage LUAD. On top of that, Kaplan-Meier-survival analysis was performed on the same patients from the GEO datasets using 4 different combination groups of recurrence risk and TNM stage: i) high risk + stage I; ii) high risk + stage II; iii) low risk + stage I; iv) low risk + stage II. Expectedly, the results showed that low risk + stage I demonstrated superior predictive capacity in RFS than the other 3 groups (Figure 6B, *P* < .0001). Altogether, these data suggest that the signature-based recurrence risk and TNM stage do complement each other in their predictive capacity on RFS of early-stage LUAD patients.

### Additional evaluation of the EMT-associated gene signature

**Figure 7.**
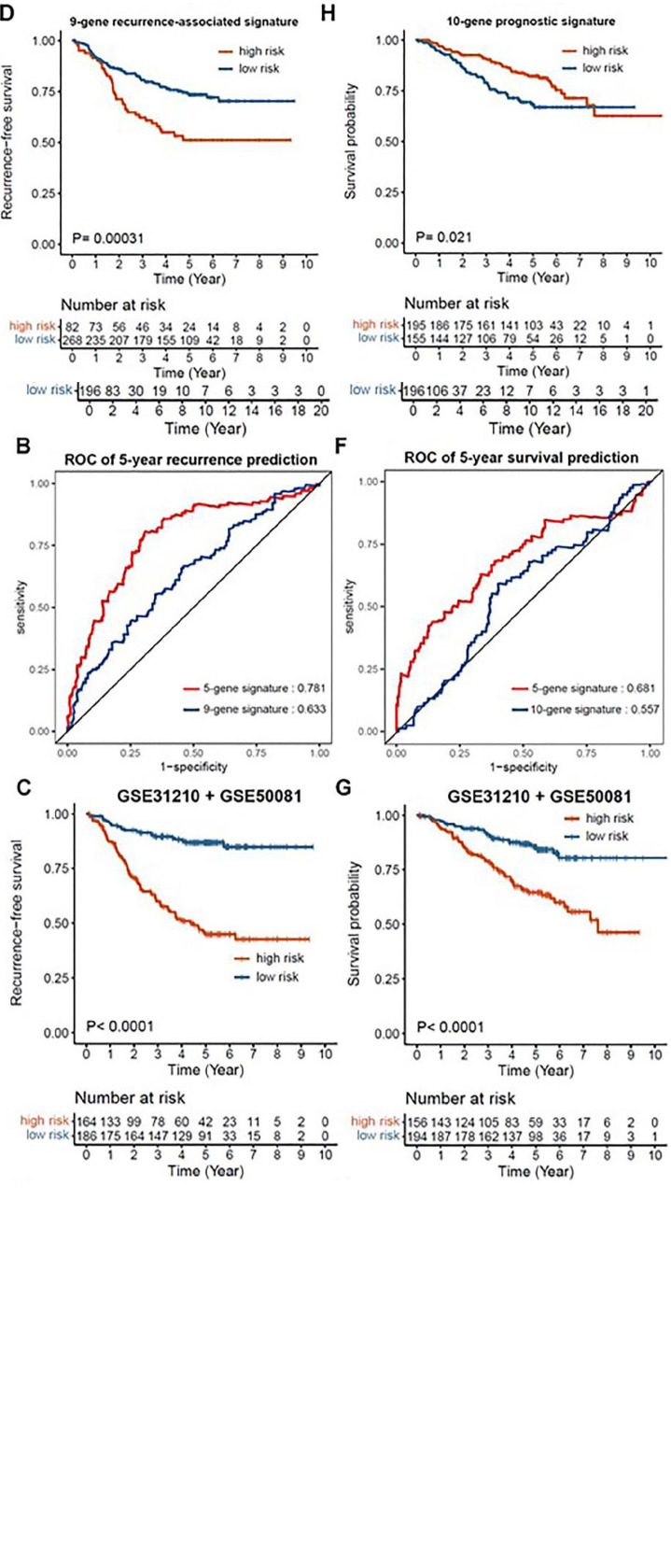
Additional evaluation of the EMT-associated gene signature: (A) Kaplan–Meier analysis of RFS in early-stage LUAD of TCGA cohort based on the risk score, (B) ROC curves for 5-year recurrence prediction of the 5-gene signature and 9-gene signature in the combined cohort of GSE31210 and GSE50081, (C and D) Kaplan–Meier analyses of RFS in the combined cohort of GSE31210 and GSE50081 based on the risk scores of the 5-gene signature and 9-gene signature, (E) Kaplan–Meier analysis of OS in early-stage LUAD of TCGA cohort based on the risk score, (F) ROC curves for 5-year survival prediction of 5-gene and 10-gene signatures in the combined cohort of GSE31210 and GSE50081, and (G and H) Kaplan–Meier analyses of OS in the combined cohort of GSE31210 and GSE50081 based on the risk scores of the 5-gene signature and 9-gene signature.

To further confirm the prognostic value of the EMT-associated gene signature, we also validated this EMT-associated signature in an external TCGA cohort. The risk scores of 307 patients with early-stage LUAD were calculated using the previous formula and were scaled between −2.139 and +2.526. The patients were subsequently stratified into the high-risk group and low-risk group based on the previous cutoff (cutoff = 0.550). Although the mRNA expression data from TCGA dataset were measured by RNA sequencing platform instead of Affymetrix platform, the EMT-associated 5-gene signature was able to effectively distinguish high-risk patients with early-stage LUAD from low-risk patients, demonstrating significant prognostic value on LUAD patients. Kaplan-Meier survival analysis showed the high-risk patients experienced a worse RFS and OS than low-risk patients with early-stage LUAD in TCGA cohort (Figure 7A and E, RFS: *P* = .041, OS: *P* = .0076 cutoff = 0.550).

Since there has been an increasing number of prognostic gene signatures being identified for LUAD, it is important to compare and evaluate the prognostic performance of different gene signatures at the same time for successful clinical application in the future. Hence, our EMT-associated gene signature was assessed by comparing with 2 other identified signatures: a 9-gene recurrence-associated signature^31^ and a 10-gene prognostic signature.^32^ The risk scores of each sample from the combined cohort of GSE31210 and GSE50081 were calculated using their formulas respectively (Supplemental Table S7) and were subsequently used for the following analyses. The ROC curve of the 5-year recurrence prediction shows that our 5-gene signature demonstrated better performance than the 9-gene recurrence-associated signature (Figure 7B, AUC = 0.781 vs AUC = 0.633, respectively). Although the 2 published signatures exhibited their predictive values in the combined cohorts, our 5-gene signature displayed more significant *p*-values in RFS (Figure 7C and D, *P*-value < .0001 vs .00031 with the cutoff of 0.851 and −0.910, respectively) and OS (Figure 7G and H, *P*-value < .0001 vs .021 with the cutoff of 0.913 and 99.820, respectively) through Kaplan–Meier analyses with their optimal cutoff values. Apart from that, in terms of the 5-year survival prediction, our 5-gene signature showed a superior prognostic value compared with the 10-gene prognostic signature (Figure 7F, AUC = 0.681 vs AUC = 0.557, respectively). Taken together, the EMT-associated 5-gene signature represented robust predictive ability in the patients with early-stage LUAD since it was successfully validated in the external TCGA cohort and demonstrated superior prognostic performance over 2 other published gene signatures.

## Discussion

The clinical outcomes of patients with early-stage LUAD vary significantly despite having similar clinical and pathological characteristics. Although the clinical guidelines recommend postoperative surveillance of LUAD patients by routine radiologic examinations such as computed tomography (CT),^33^ small tumors remain undetectable due to the limitations of current clinical management approaches. Therefore, a novel and robust tool is urgently needed to predict and screen for the potential recurrence of early-stage LUAD after surgical resection. Numerous studies have demonstrated the critical role of EMT in tumor initiation and progression, especially in the advanced stage with metastasis. However, tumor cells in the early stage have undergone EMT and resulted in dissemination in the form of micrometastasis or occult tumor cells. Moreover, during the EMT process, the resulting mesenchymal cells acquire the ability to evade immune surveillance^34^ and resisting to therapeutic regimens, such as chemotherapeutics, radiotherapy, targeted treatment and immunotherapy, which consequently allow for the survival of isolated tumor cells and the formation of minimal residual disease (MRD) in cancer patients. These may help explain why the patients carry a high risk of tumor recurrence despite receiving radical resection and adjuvant therapies. Hence, it appears clear that EMT is fundamentally linked to tumor recurrence.

In the present study, we identified that EMT displayed strong positive correlations with angiogenesis, hypoxia, coagulation, KRAS and TGFβ signaling pathways in the patients with early-stage LUAD, which correspond to other publications on the role of EMT in several types of carcinomas such as breast cancer, gastric cancer, lung cancer.^10,35-39^ Besides EMT, apoptosis is another fundamental event that is tightly linked to the physiological and disease-related cellular processes. Interestingly, the modulation of EMT and apoptosis is largely dependent on the functions of TGFβ.^40^ Although the current notion of EMT is mainly associated with resistance to apoptosis,^41^ we found that EMT was positively correlated with apoptosis in the patients with early-stage LUAD, which can be supported by a publication showing the simultaneous occurrence of EMT and apoptosis triggered by TGFβ1.^42^ It has been identified that TGFβ induced EMT in both unsynchronized cells and those synchronized at the G1/S phase of the cell cycle, while TGFβ-induced apoptosis occurred only at the G2/M phase of the cell cycle, indicating the possibility of simultaneous occurrence of EMT and apoptosis.^43^ Since tumor cells are highly heterogenous with cells at different phases of the cell cycle, it is likely that induction of EMT leads to an increase in apoptosis levels. However, the overall net effect is determined by the cellular context and the specific state of the cells.^43^

Apart from that, our analysis also revealed that EMT was negatively associated with the tumor purity of LUAD. This reflects the fact that most of the gene expression profiling is performed with bulk clinical specimens that contain a mix of tumor and stromal cells. Since stromal cells, such as fibroblasts, contain EMT canonical markers,^44^ it may explain our finding that higher levels of EMT occurred when the tumor purity was low. In line with the presence of fibroblasts, this study also demonstrated that patients with early-stage LUAD displayed EMT that was strongly correlated to TME cells and immune checkpoint genes, which are the potential targets for targeted therapies and immunotherapies. For example, EMT showed a remarkably high positive correlation with fibroblasts, which are known to play important roles in the EMT process in the tumor microenvironment.^45^ Several clinical trials related to interference with cancer-associated fibroblasts (CAF) activation, CAF action and CAF normalization are currently underway,^46^ involving targeting TGFβ, C-X-C motif chemokine receptor 4 (CXCR4) or vitamin A metabolism. Moreover, our analyses revealed correlations between EMT and immune checkpoints in early-stage LUAD, such as CD200, TNFSF4 and SIRPA, which have also been demonstrated in other types of carcinomas. For instance, CD200 has been shown to induce EMT in head and neck squamous carcinoma through β-catenin-mediated nuclear translocation.^47^ A pan-cancer analysis involving 1934 tumors demonstrated high expression of the *TNFSF4* gene (also known as OX40L) in tumors with the most mesenchymal EMT scores.^48^ Apart from that, it was also identified that CD47-SIRPα signaling induced EMT and cancer stemness, and was linked to a poor prognosis in patients with oral squamous carcinoma.^49^ In addition, this study found that EMT was significantly related to poor RFS and OS in patients with early-stage LUAD. Our findings showed the multi-faceted roles of EMT in patients with early-stage LUAD, which may provide a series of novel targets and strategies for early-stage LUAD treatment.

Subsequently, we identified an EMT-associated 5-gene signature based on WGCNA, LASSO method and Cox proportional hazards regression model in the GEO database. The signature comprised of *AGL, ECM1, ENPP1, SNX7*, and *TSPAN12* that could predict the recurrence of patients with early-stage LUAD. Recent studies have demonstrated that ECM1, ENPP1, TSPAN12 were involved in tumor invasion and metastasis. For instance, in breast cancer, *ECM1* gene overexpression induced EMT progression via regulating the beta-catenin signaling pathway and the expression levels of ECM1 protein in patient plasma were associated with the tumor recurrence.^50^ Furthermore, ECM1 protein has also been found to be highly expressed in hepatocellular carcinoma and the overexpression of ECM1 protein resulted in the promotion of migration and invasion of tumor cells via EMT induction.^51^ As for the *ENPP1* gene, it has been reported that *ENPP1*-knockdown reduced the expression of cancer stem cell markers and reversed TGFβ-induced EMT phenotypes in non-small cell lung cancer, including cell migration, the repression of E-cadherin and induction of vimentin.^52^ Lastly, the role of the *TSPAN12* gene in lung cancer has also been demonstrated. A study revealed that knocking down TSPAN12 could promote lung cancer cell proliferation and migration; moreover, the *TSPAN12* gene was significantly down-regulated in NSCLC tissues compared with their matched normal adjacent tissues,^53^ indicating that TSPAN12 was a favorable factor during NSCLC progression. It is noteworthy that the relationship of EMT with *AGL* and *SNX7* has not been reported previously, and therefore further investigation is needed. In short, these 5 genes appear to be closely related to EMT and thus tumor recurrence. This gene signature could successfully predict the recurrence of early-stage LUAD in both training and validation cohorts. Moreover, this EMT-associated signature was independent of the clinical characteristics, including age, gender, smoking and TNM stage. Our present study showed that this EMT-associated signature could serve as an independent prognostic factor for the recurrence of early-stage LUAD after considering other clinical and pathologic factors. Moreover, the combination of signature-based recurrence risk and TNM stage could better predict the recurrence of early-stage LUAD. In particular, it was demonstrated that the patients with low recurrence risk and in stage I had the best clinical outcome in terms of RFS compared with other patients with high risk and/or stage II. In addition, the superior predictive values on RFS and OS were represented in our EMT-associated 5-gene signature compared with the other 2 identified signatures when evaluated in the combined cohort of GSE31210 and GSE50081.

Collectively, our EMT-associated 5-gene signature is a promising predictive tool for the recurrence of early-stage LUAD. In this study, GSE31210 and GSE50081 datasets from the same platform were used and the batch effects between them were removed to ensure the signature was more reliable and accurate. This is the first study to establish an EMT-associated gene signature for the patients with early-stage LUAD, with a good predictive performance and clinical independence in the training cohort and validation cohorts. Applying this 5-gene signature to clinical settings is relatively easy since it could be performed with simple molecular biology techniques such as quantitative real-time polymerase chain reaction (PCR). There are also limitations to our study. Although a wide range of correlation analyses of EMT phenotype was conducted, some underlying molecular mechanisms remained elusive. Further experiments are required to unravel the EMT-associated mechanisms in the recurrence of early-stage LUAD. Moreover, due to the lack of clinically relevant information in the cohorts, the impact of neoadjuvant and adjuvant therapies as well as lymph node metastasis on our signature could not be analyzed, which are also critical factors for affecting recurrence. Hence, further prospective and large-scale clinical studies are warranted to validate our findings.

## Conclusions

Taken together, our present study showed the diverse EMT states of the patients with early-stage LUAD, providing us a better understanding of the roles of EMT in tumor progression and recurrence. Moreover, the robust EMT-associated 5-gene signature that had been established and validated in this study could be highly predictive for the recurrence among the patients with early-stage LUAD, which may also bring novel strategies for postoperative monitoring and individualized treatment to the patients with early-stage LUAD.

## Supplemental Material

sj-docx-1-cix-10.1177_11769351221100727 – Supplemental material for An In Silico Analysis Reveals an EMT-Associated Gene Signature for Predicting Recurrence of Early-Stage Lung Adenocarcinoma Click here for additional data file. Supplemental material, sj-docx-1-cix-10.1177_11769351221100727 for An In Silico Analysis Reveals an EMT-Associated Gene Signature for Predicting Recurrence of Early-Stage Lung Adenocarcinoma by Yi Han, Fang Cheng Wong, Di Wang and Christoph Kahlert in Cancer Informatics
