# Supplementary material for: An In Silico Analysis Reveals an EMT-Associated Gene Signature for Predicting Recurrence of Early-Stage Lung Adenocarcinoma
Source: Cancer Inform. 2022 May 23;21:11769351221100727. doi: 10.1177/11769351221100727 (PMC9133999; doi:10.1177/11769351221100727)
Supplement: sj-docx-1-cix-10.1177_11769351221100727 – Supplemental material for An In Silico Analysis Reveals an EMT-Associated Gene Signature for Predicting Recurrence of Early-Stage Lung Adenocarcinoma [file sj-docx-1-cix-10.1177_11769351221100727.docx]

**An *in silico* Analysis Reveals an EMT-Associated Gene Signature for Predicting Recurrence of Early-Stage** **Lung Adenocarcinoma**

**Supplementary Materials**

**Table S1.** Patients’ features for the training and validation cohorts

|  | **Training cohort** | **Validation cohort** |
| --- | --- | --- |
| **GEO dataset** | GSE31210 | GSE50081 |
| **Platform** | Affymetrix Human Genome U133 Plus 2.0 Array | Affymetrix Human Genome U133 Plus 2.0 Array |
| **Number of samples** | 226 | 124 |
| **Age, years** |  |  |
| Median | 61 | 70 |
| Range | 30-76 | 40-86 |
| **Gender** |  |  |
| Male | 105 | 63 |
| Female | 121 | 61 |
| **Smoking** |  |  |
| Non-smoker | 115 | 23 |
| smoker | 111 | 90 |
| NA | 0 | 11 |
| **LUAD stage** |  |  |
| Stage I | 168 | 90 |
| Stage II | 58 | 34 |
| **Follow-up time, years** |  |  |
| Median | 4.47 | 3.73 |
| Range | 0.16-9.50 | 0.09-8.26 |

^#^NA, not available; LUAD, lung adenocarcinoma

**Table S2**. 31 immune checkpoint molecules

| **Family** | **Immune checkpoint molecules** | **Gene symbol** | **References** |
| --- | --- | --- | --- |
| **B7 subfamily** | PD-L1 | CD274 | [23], [24], [25] |
|  | B7-H3 | CD276 | [23], [24], [25] |
|  | B7-1 | CD80 | [23], [24], [25] |
|  | B7-2 | CD86 | [23], [24], [25] |
|  | CTLA4 | CTLA4 | [23], [24] |
|  | HHLA2 | HHLA2 | [23] |
|  | ICOS | ICOS | [23], [24] |
|  | ICOSLG | ICOSLG | [23], [24] |
|  | PD1 | PDCD1 | [23], [24], [25] |
|  | PD-L2 | PDCD1LG2 | [23], [24] |
|  | B7-H4 | VTCN1 | [23], [25] |
| **Immunoglobulin family** | VISTA | VSIR | [23], [24] |
|  | TMIGD2 | TMIGD2 | [23] |
|  | CD200 | CD200 | [26] |
|  | CD200R1 | CD200R1 | [26] |
|  | CD28 | CD28 | [23], [24], [25] |
|  | CD47 | CD47 | [24], [25] |
|  | LAG3 | LAG3 | [23], [24] |
|  | SIRPα | SIRPA | [24], [25] |
|  | TIM3 | HAVCR2 | [23], [24], [25] |
| **TNF superfamily** | BTLA | BTLA | [23] |
|  | CD27 | CD27 | [23], [24], [25] |
|  | CD40 | CD40 | [23], [25] |
|  | CD40LG | CD40LG | [23], [25] |
|  | CD70 | CD70 | [23], [24], [25] |
|  | GITR | TNFRSF18 | [23], [24] |
|  | OX40 | TNFRSF4 | [23] |
|  | OX40L | TNFSF4 | [23] |
|  | 4-1BB | TNFRSF9 | [23] |
|  | 4-1BBL | TNFSF9 | [23] |
| **IDO synthase** | IDO1 | IDO1 | [23], [24] |


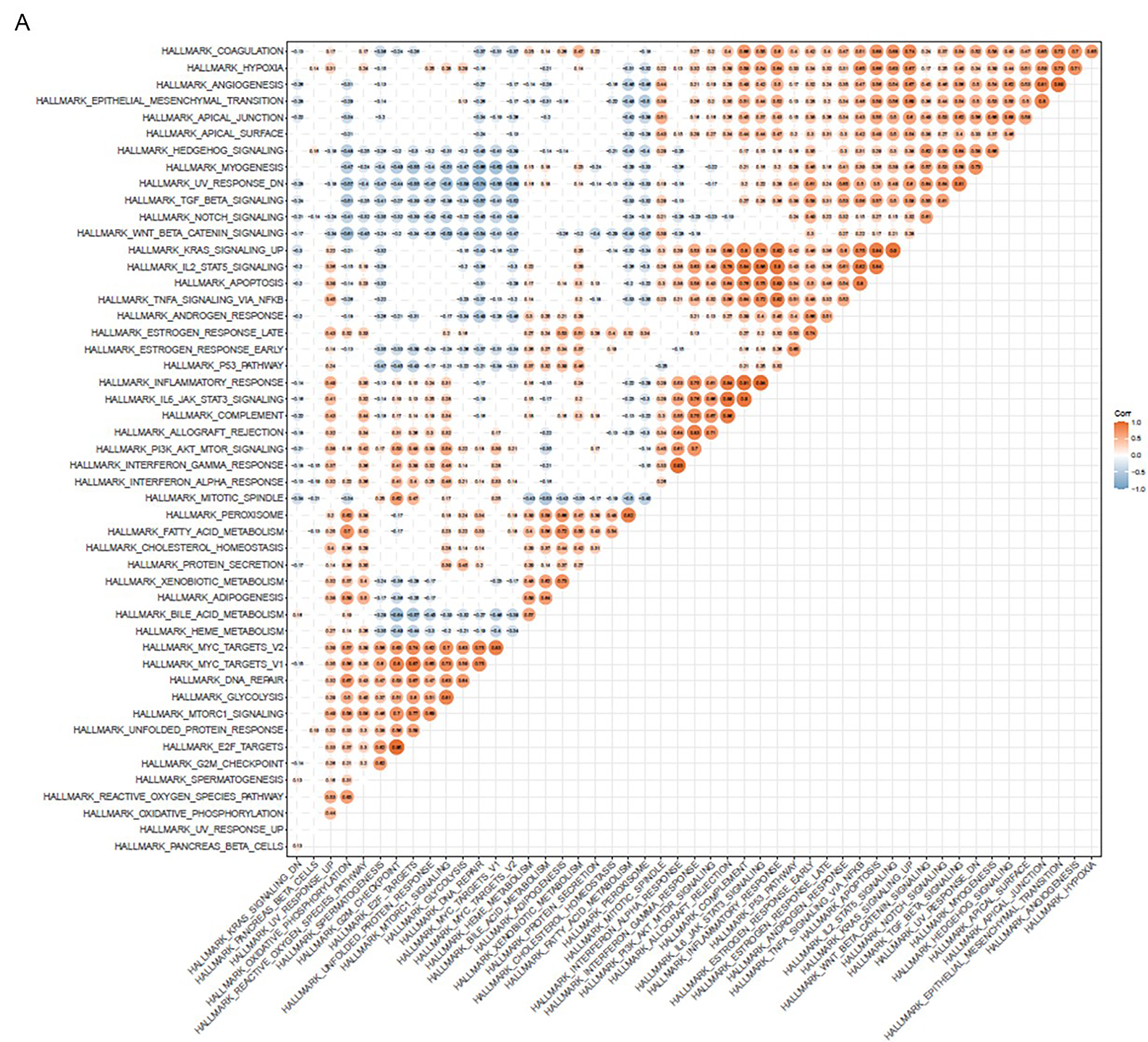


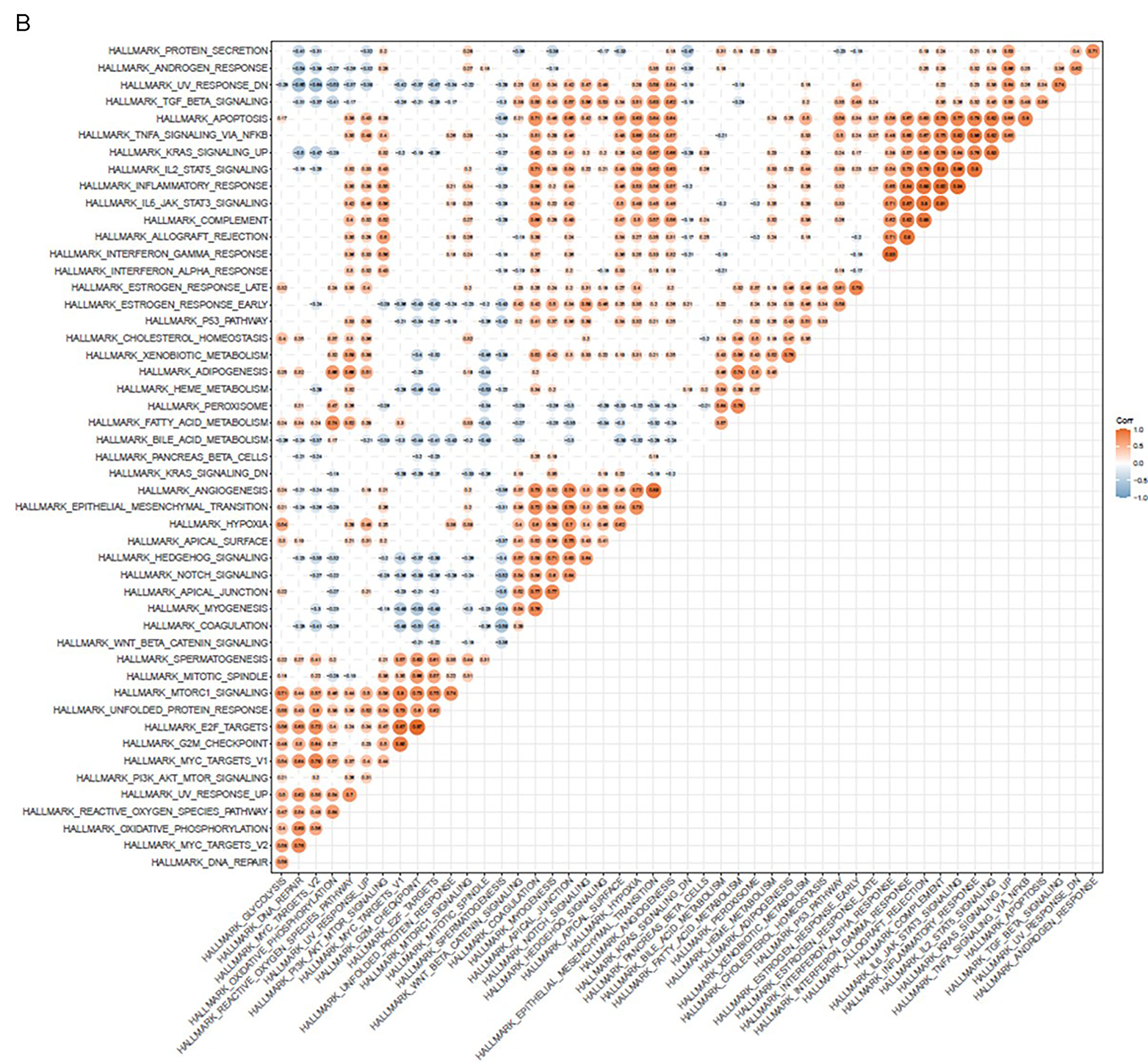


**Figure S1.** The Pearson correlations between 50 HALLMARK gene sets in GSE31210 (A) and GSE50081 (B) with *p*<0.05.

**
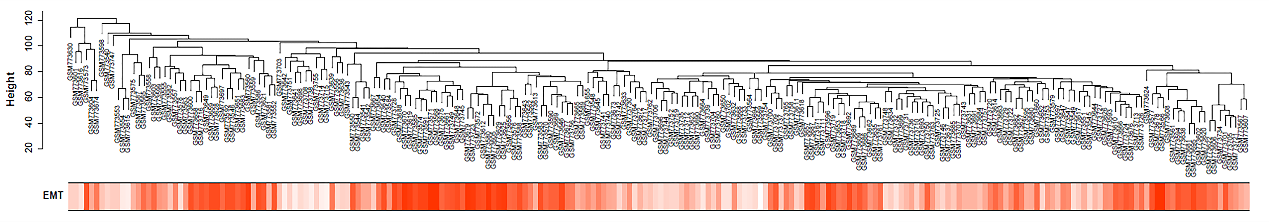
**

**Figure S2.** Clustering dendrogram of 226 samples in GSE31210.


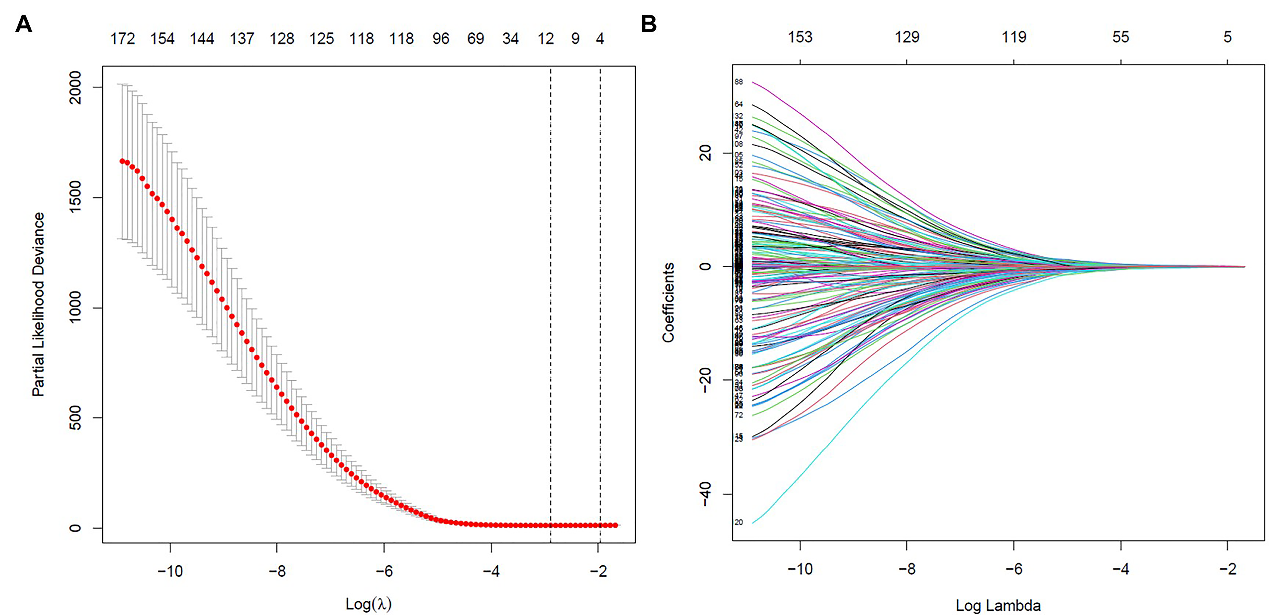


**Figure S3.** LASSO regression analysis. (A) Optimal lambda was selected using 10-fold cross-validation via minimum criteria. Dotted vertical lines represent the optimal lambda (λ) values with the minimum criteria (left) and the 1 standard error of the minimum criteria (right). (B) LASSO coefficient profiles of the 187 variables were plotted against the log (lambda) sequence.
